# Characteristics and treatment outcomes of portal hypertension after living donor liver transplantation

**DOI:** 10.1007/s00595-025-03222-8

**Published:** 2026-01-07

**Authors:** Atsuyoshi Mita, Yasunari Ohno, Yuichi Masuda, Koji Kubota, Tsuyoshi Notake, Akira Shimizu, Yuji Soejima

**Affiliations:** Division of Gastroenterological, Hepato-Bilianry-Pancreatic, Trasplantation and Pediatric Surgery, Department of Surgery, Shinshu University School of Medicine, 3-1-1 Asahi, Matsumoto, 390-8621 Japan

**Keywords:** Post-transplant portal hypertension, Liver transplantation, Interventional radiology, Re-transplantation, Splenectomy

## Abstract

**Purpose:**

Portal hypertension (PoH) after liver transplantation is a severe complication that results in graft loss. We investigated the characteristics and evaluated the treatment outcomes of PoH after living donor liver transplantation (LDLT).

**Methods:**

This single-center, retrospective cohort study included 325 LDLT recipients.

**Results:**

Of the subjects, 37 (11.4%) had a PoH. The 10- and 20-year graft survival rates were significantly lower in patients with PoH than in those without PoH (69.1% vs. 90.8% and 42.1% vs. 84.7%, respectively; *p* < 0.0001). The types of PoH were pre-hepatic, hepatic, and post-hepatic in 16, 13, and 8 patients, respectively. Interventional radiology was performed for PoH in all post-hepatic PoH patients and in 62.5% of pre-hepatic PoH patients. Notably, 46.2% of the patients with hepatic PoH required re-transplantation. The 10-year graft survival rate was significantly worse in patients with hepatic PoH than in those with pre- and post-hepatic PoH (46.2% vs. 86.7% and 75.0%, respectively; *P* < 0.05). Post-transplant PoH was an independent predictor of graft loss after LDLT (hazard ratio, 5.73; 95% confidence interval: 2.43–13.55, *P* < 0.0005).

**Conclusions:**

Post-transplant PoH negatively affected the graft survival in LDLT recipients. Pre-hepatic, hepatic, and post-hepatic PoH cases had different characteristics, requiring different treatments. Therefore, an appropriate diagnosis and treatment are important.

**Supplementary Information:**

The online version contains supplementary material available at 10.1007/s00595-025-03222-8.

## Introduction

In portal hypertension (PoH), vascular resistance increases due to occlusion of the portal vein (PV) (pre-hepatic), hepatic sinusoids (hepatic), and hepatovenous outflow (post-hepatic) inside and outside the liver, resulting in abnormal PV blood circulation, such as increased PV pressure above 5–10 mmHg [1]. The Japanese guidelines define PoH as an increased portal pressure above 200 mmH_2_O or 14.7 mmHg. Liver cirrhosis is the most common cause of PoH and accounts for most liver transplantation (LT) indications [2]. 

Conversely, PoH after LT is rare, with a reported frequency of 2.8%; however, it is a severe complication resulting in graft loss [3]. Post-transplant PoH manifests as a variety of symptoms similar to pre-transplant end-stage liver disease, including variceal bleeding, refractory ascites, pleural effusion, splenomegaly, and hepatic encephalopathy [4]. In addition to hepatic disorders similar to pre-transplantation, vascular disorders associated with surgical procedures or immune reactions play a crucial role in causing post-transplant PoH. The wide variety of treatment procedures further contributes to the complexity of managing PoHs [5]. However, owing to limited reports, the diagnosis and treatment of PoH after LT are uncertain.

The present study clarified the characteristics and evaluated the treatment outcomes of PoH after LT.

## Methods

### Study design

This single-center retrospective cohort study was conducted at a university hospital in Japan.

Overall, 341 patients underwent living donor liver transplantation (LDLT) at Shinshu University Hospital between June 1990 and March 2023. First, 16 patients who lost their liver graft within 3 months after LT were excluded from this study because the impact of pre-transplant PoH should be reduced in their post-transplant condition and treatment. The causes of death in these patients were infection in four, a poor graft function in three, multiple organ failure in two, respiratory failure in two, and hepatic artery thrombosis, cerebral bleeding, thrombotic thrombocytopenic purpura, veno-occlusive disease, and viral-associated hemophagocytic syndrome in one patient each. Ultimately, 325 patients were included in this study.

Post-transplant PoH was diagnosed based on the following complications that required any treatment: gastrointestinal varices with or without bleeding, refractory hepatic hydrothorax and/or ascites that could not be explained as a surgical complication, hepatic encephalopathy, pulmonary hypertension, and developing hypersplenism. The PV pressure was not measured in any patient because it was an invasive procedure.

The patients were categorized into PoH and no-PoH groups that included patients with (*n* = 37) and without (*n* = 288) post-transplant PoH, respectively. The primary outcomes were the graft loss and survival rates. In addition, we evaluated the treatment outcomes for post-transplant PoH as sub-analyses in the following types of PoH according to their origin: pre-hepatic, hepatic, and post-hepatic PoH (*n* = 16, 13, and 8, respectively).

### Graft selection

Left hemi-liver and lateral segment grafts were selected in LDLT based on the preoperatively calculated graft-volume-to-standard-liver-volume ratio (GV/SLV) [6]. Right hemi-liver graft was considered when the left hemi-liver graft was too small to meet the preoperatively calculated GV/SLV ≥ 30% and the ratio of remnant living donor liver volume to total donor liver volume was ≥ 35%.

### Treatment procedure

**Fig. 1:**
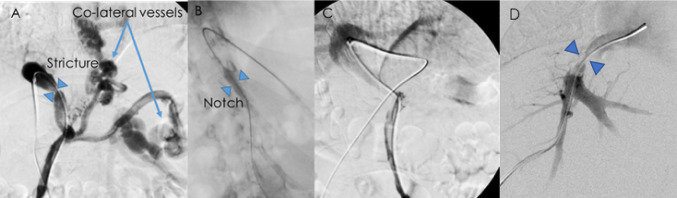
Procedure of percutaneous transhepatic portal vein angioplasty and percutaneous transhepatic hepatic vein angioplasty (PTHA). a: The portal vein is punctured using ultrasonography-guided percutaneous transhepatic method. A catheter was then inserted into the portal vein. Portography showing an anastomotic stricture (arrowheads) and collateral vessels (arrows). The catheter was inserted into the main trunk of the portal vein, and the portal vein pressure was measured on both sides of the stricture, providing a diagnosis of portal hypertension based on a pressure gradient. b: A balloon catheter is inserted through the anastomotic stricture using the guidewire technique. The stricture was dilated using balloons, starting from a small diameter, and then extended step by step until the notch vanished (arrowheads). We avoided using vascular stents because of the difficulties associated with their removal. c: Portal vein pressure on both sides of the dilated point was remeasured, and the catheter was removed. The hepatic parenchyma was filled with a gelatin sponge for hemostasis. d: The hepatic vein was punctured using ultrasonography-guided percutaneous transhepatic method when performing PTHA. Venography shows an anastomotic stricture (arrowheads)

Anastomotic stricture of the PV and hepatic vein (HV) was initially treated via interventional radiology (IVR) (Fig. 1). Percutaneous transhepatic PV angioplasty (PTPoA) was performed. The catheter was inserted into the PV of the liver graft after puncturing using an ultrasound-guided percutaneous transhepatic technique. It was passed through the anastomosis using the guidewire technique with a short vascular sheath to confirm anastomotic stricture by direct portography (Fig. 1a). The catheter was exchanged with a balloon catheter (Bandicoot RX; Kaneka Medical Products, Osaka, Japan), and the balloons were inflated at 14 atm for 30 s each (Fig. 1b). Subsequently, the balloon diameter was increased from 3 to 5 mm, depending on the PV diameter. The postangioplasty portogram revealed resolution of the stricture and marked flow reduction from the collateral vessel (Fig. 1c). Next, the punctured tract was embolized using gel foam when the vascular access sheath was removed.

HV angioplasty was performed similarly to PTPoA, using the percutaneous transhepatic or transjugular vein approach. A catheter was inserted and passed through the anastomosis between the inferior vena cava (IVC) and HV in the liver graft (Fig. 1d). Balloon dilatation was performed for strictures of the HV, as described above. Low-molecular-weight heparin was administered at one to two weeks for postprocedural anticoagulation, followed by warfarin or direct oral anticoagulants at six months.

Ascites drainage (large-volume paracentesis) was performed to relieve abdominal distension and/or for diagnostic purposes. In most patients, a temporary drainage tube was inserted into the abdomen under local anesthesia. In contrast, cell-free and concentrated ascites reinfusion therapy was administered in other patients.

Re-transplantation was performed for graft failure if the recipient was able to obtain a liver graft from a living or deceased donor. However, the number of re-transplantation cases was limited because of donor shortage. Many patients with graft failure due to post-transplant PoH desired deceased donor liver transplantation (DDLT) but died in the absence of an appropriate graft.

### Statistical analyses

Continuous variables are presented as the mean ± standard deviation for normally distributed data or as median (interquartile range [IQR]) for non-normally distributed data. Categorical variables were presented as proportions. Patient characteristics were compared using Student’s *t*-test or the Mann–Whitney U test for continuous variables and chi-squared or Fisher’s exact test for categorical variables.

Graft survival rates after LT were analyzed using the Kaplan–Meier method with Cox’s proportional hazards model, and the difference was evaluated using a log-rank test. Patient death during the maintenance of the liver graft function was treated as a sensor in the graft survival rate analysis. Cox regression was performed to identify predictive factors, including the recipient age at LT, sex, presence of hepatocellular carcinoma (HCC), model of end-stage liver disease (MELD) score, Child-Pugh score, GV/SLV, ABO incompatibility, donor age, intraoperative portal flow modulation, rejection episodes, post-splenectomy, and post-transplant PoH for graft loss after LDLT. Factors associated with graft loss were adjusted for confounding factors using multivariate Cox proportional hazards models to identify the independent predictors of graft loss. The results are expressed as hazard ratios (HRs) with 95% confidence intervals (CIs).

All statistical tests were 2-sided, and statistical significance was set at *P* < 0.05. All statistical analyses were performed using the JMP software program, version 18 (SAS Institute, Cary, NC, USA).

## Results

### Patient demographics

Of the 325 LDLT recipients, 37 (11.4%) were diagnosed with PoH 3.8 years (IQR: 1.0, 8.5) after LT (PoH group). The first symptoms included gastrointestinal varices with or without bleeding, hepatic hydrothorax and/or ascites, developing hypersplenism, and others in 18, 9, 3, and 7 patients, respectively. The median age of the patients was 15.1 years at LT, with 54.1% being pediatric patients (< 18 years old at LT). More than half of the patients were female (56.8%). Patients ≤ 12 years old at LT had a median pediatric end-stage liver disease score of 16, whereas those > 12 years old had a mean MELD score of 15. These scores were comparable to those of 288 patients without PoH (no-PoH group; Table 1). In addition, the primary disease for LT was comparable between the two groups. Post-transplant PoH also occurs in LT recipients with non-cirrhotic metabolic diseases, such as citrullinemia. Graft volume, GV/SLV, and graft recipient weight ratio were comparable between the two groups. Intraoperative portal flow modulation, including splenectomy and splenic artery ligation, was less frequently performed in the PoH group than in the non-PoH group (2.7% vs. 14.6%, *P* = 0.02). *P*, including cases performed in the pre-transplant period (Table 1).

**Table 1:** Characteristics of patients in the PoH and no-PoH groups

Child-Pugh score	PoH, *n*=37	no-PoH, *n*=288	*p*-value
Age at LT, years	15.1 (1.3, 42.1)	31.5 (2.5, 52.9)	0.054
Pediatrics, n (%)	20 (54.1)	110 (38.2)	0.07
Female, n (%)	21 (56.8)	166 (57.6)	0.92
Primary disease, n (%)			0.69
Biliary disease	20 (54.1)	136 (47.2)	
Hepatocellular disease	11 (29.7)	98 (34.0)	
Metabolic liver disease	6 (16.2)	50 (17.4)	
Others	0 (0.0)	4 (1.4)	
HCC, n (%)	2 (5.4)	44 (15.3)	0.07
Child-Pugh score			
PELD score (*n*=115)	16 (11.5, 23)	16.5 (10.3, 23)	0.76
MELD score (*n*=210)	15 (10, 20)	14 (9, 19)	0.34
Graft types, n (%)			0.47
Lateral segment	13 (35.1)	94 (32.6)	
Left lobe	23 (62.2)	172 (59.7)	
Right lobe	1 (2.79)	22 (7.6)	
Graft volume, mL	360.0 (295.5, 422.0)	382 (287.0, 449.0)	0.33
GV/SLV, %	45.0 (36.9, 83.0)	45.2 (36.8, 72.4)	0.42
GRWR, %	1.02 (7.7, 2.82)	0.95 (0.73, 2.30)	0.30
Relationship to donor, n (%)			0.057
Parents	22 (59.5)	139 (48.9)	
Children	4 (10.8)	74 (26.1)	
Non-kinship	3 (8.1)	38 (13.4)	
Others	8 (21.6)	33 (11.6)	
ABO-compatibility, n (%)			0.34
Identical	29 (78.4)	209 (72.6)	
Compatible	5 (13.5)	65 (22.6)	
Incompatible	3 (8.1)	14 (4.9)	
Intraoperative portal flow modulation	1 (2.7)	42 (14.6)	0.02
Operation time, min.	885 (714, 947)	867 (731, 1012)	0.63
Blood loss, mL	800 (400, 3570)	1390 (598, 3808)	0.052
Post-splenectomy, n (%)	1 (2.7)	42 (14.6)	0.02

GRWR, graft recipient weight ratio; GV/SLV, ratio of graft volume to standard liver volume; HCC, hepatocellular carcinoma; LT, liver transplantation; MELD, model of end-stage liver disease; PELD, pediatric end-stage liver disease; PoH, portal hypertension.

We separately evaluated the characteristics of the pediatric (< 18 years old at LT, *n* = 130) and adult (*n* = 195) cohorts (Supporting Information, Tables 1 and 2). Pediatric patients experienced PoH more frequently after LDLT than adult patients (16.2% vs. 8.2%, *P* < 0.05).

**Fig. 2:**
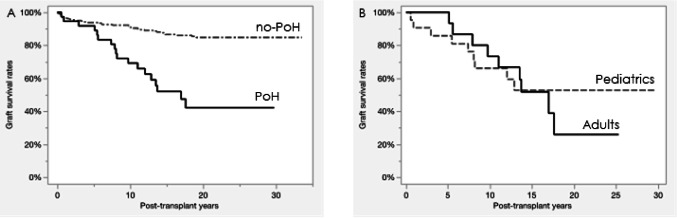
Graft survival rates after liver transplantation (LT). Graft survival rates after LT were depicted using the Kaplan–Meier method with Cox’s proportional hazards model. (a) The graft survival rate of LT recipients with portal hypertension (PoH) (PoH group, *n* = 37, solid line) was significantly lower than that of those without PoH (no-PoH group, *n* = 288, long dashed-dotted line) (*p* < 0.0001). The 10-year survival rates in the PoH and non-PoH groups were 69.1% and 90.8%, respectively. The 20-year survival rates in the PoH and non-PoH groups were 42.1% and 84.7%, respectively. (b) The post-transplant graft survival rate was comparable between adult and pediatric recipients with post-transplant PoH. The 10- and 20-year graft survival rates were 73.3% and 25.9%, respectively, in adults, and 66.0% and 52.8%, respectively, in pediatric patients (*p* = 0.70)

According to the Kaplan–Meier method with Cox’s proportional hazards model, the graft survival rate was significantly lower in the PoH group than in the non-PoH group (10-year graft survival rate, 69.1% vs. 90.8%; 20-year graft survival rate, 42.1% vs. 84.7%; *P* < 0.0001, Fig. 2A). Recipients with post-transplant PoH showed comparable graft survival rates in both the adult and pediatric cohorts (10-year graft survival rate, 66.0% vs. 73.3%; 20-year graft survival rate, 52.8% vs. 25.9%; *P* = 0.70, Fig. 2B).

**Table 2:** Patient characteristics in the three types of PoH

Variables	Hepatic *n*=13	Pre-hepatic *n*=16	Post-hepatic *n*=8	*p*-value
Age at LT, years	15.2 (8.6, 37.3)	15.7 (1.2, 45.5)	1.6 (0.9, 26.3)	0.32
Pediatrics, n (%)	7 (53.9)	8 (50.0)	6 (75.0)	0.47
Female, n (%)	8 (61.5)	10 (62.5)	3 (37.5)	0.46
Primary disease				0.24
Biliary disease	6 (46.2)	11 (68.8)	3 (37.5)	
Hepatocellular disease	6 (46.2)	2 (12.5)	3 (37.5)	
Metabolic liver disease	1 (7.7)	3 (18.8)	2 (25.0)	
MELD score	19.5 (15, 38.5) *n*=10	14.5 (10.3, 17.5) *n*=8	7 (7, 7) *n*=2	<0.05
PELD score	11 (4, 23) *n*=5	19.5 (15, 24.5) *n*=6	16 (10.3, 23.8) *n*=6	0.35
Child–Pugh score	10 (7, 11.5)	9.5 (8.3, 10.8)	9.5 (5.8, 12.8)	0.96
Donor, n (%)				0.57
Parents	10 (76.9)	9 (56.3)	5 (62.5)	
Children	1 (7.7)	2 (12.5)	0 (0.0)	
Non-kinship	0 (0.0)	1 (6.3)	0 (0.0)	
Others	2 (15.4)	4 (25.0)	3 (37.5)	
Donor age, years	42.4 ± 8.2	35.2 ± 10.0	39.6 ± 10.1	0.13
Female donor, n (%)	8 (61.5)	4 (25.0)	4 (50.0)	0.12
Graft type, n (%)				0.055
Lateral segment	2 (15.4)	6 (37.5)	6 (75.0)	
Left lobe	20 (77.0)	10 (62.5)	2 (25.0)	
Right lobe	1 (7.7)	0 (0.0)	0 (0.0)	
Graft volume, g	373.2 ± 108.8	357.89 ± 65.0	326.1 ± 76.7	0.47
GV/SLV, %	43.1 (38.1, 58.8)	46.7 (34.5, 115.7)	(52.1, 102.1)	0.13
GRWR, %	0.87 (0.76, 1.65)	1.05 (0.75, 4.76)	2.71 (1.27, 3.54)	0.13
ABO blood type compatibility, n (%)				0.30
Identical	11 (84.6)	11 (68.8)	8 (100.0)	
Compatible	1 (7.7)	3 (18.8)	0 (0.0)	
Incompatible	1 (7.7)	2 (12.5)	0 (0.0)	
Interval between LT and developing PoH, years	7.9 (3.4, 14.5)	3.3 (0.9, 4.4)	0.7 (0.1, 5.0)	<0.01
Symptom, n (%)				
Acites and/or pleural effusion	10 (76.9)	6 (37.5)	7 (87.5)	0.02
Esophagogastric varices	8 (61.5)	11 (68.8)	1 (12.5)	0.02
Portal vein thrombosis	6 (46.2)	8 (50.0)	0 (0.0)	0.011
Treatment, n (%)				<0.001
Drainage of pleural and/or ascites	2 (15.4)	2 (12.5)	0 (0.0)	
endoscopy	2 (15.4)	2 (12.5)	0 (0.0)	
IVR	1 (7.7)	10 (62.5)	8 (100.0)	
Surgical intervention	2 (15.4)	3 (18.8)	0 (0.0)	
Re-transplantation	6 (46.2)	1 (6.3)	0 (0.0)	
Follow-up, year	15.8 ± 6.6	16.3 ± 8.4	11.5 ± 7.6	0.34

GRWR, graft recipient weight ratio; GV/SLV, ratio of graft volume to standard liver volume; IVR, interventional radiology; LT, liver transplantation; MELD, model of end-stage liver disease; PELD, pediatric end-stage liver disease; PoH, portal hypertension.

### Types of PoH after LT

The PoH group included 16, 13, and 8 patients with pre-hepatic, hepatic, and post-hepatic PoH, respectively. Primary diseases for LT were comparable among the three groups (*P* = 0.24) (Table 2). Pre- and post-hepatic PoH occurred after LT for non-cirrhotic metabolic liver diseases, such as familial amyloid polyneuropathy and citrullinemia. The median MELD score was significantly different among the three groups (14.5, 19.5, and 7 in the pre-hepatic, hepatic, and post-hepatic PoH groups, respectively; *p* < 0.05). The donor factors were comparable among the three groups. Post-transplant PoH developed earliest in the post-hepatic PoH group, followed by the pre-hepatic and hepatic PoH groups (0.7, 3.3, and 7.9 years after LT, respectively; *P* < 0.01). Esophagogastric varices and PV thrombosis (PVT) occurred more frequently in the hepatic and pre-hepatic PoH groups than in the post-hepatic PoH group (66.7% vs. 71.4% vs. 0.0%, respectively; *p* < 0.05; and 50.0% vs. 46.2% vs. 0.0%, respectively; *P* < 0.05). Ascites and/or pleural effusion occurred more frequently in the hepatic and post-hepatic PoH groups than in the pre-hepatic PoH group (76.9% and 87.5% vs. 37.5%, respectively; *P* < 0.05).

Among the 13 patients with hepatic PoH, pathological findings included liver cirrhosis due to recurrent primary disease, fibrosis associated with rejection, cholestatic cirrhosis after biliary complication, and sinusoidal objective disease in 5, 5, 2, and 1, respectively.

IVR was performed for PoH in all patients in the post-hepatic PoH group and in 62.5% of those in the pre-hepatic PoH group (Table 2). In contrast, 46.2% of the patients in the hepatic PoH group required re-transplantation. Among the 13 patients in the hepatic PoH group, 6 (46.2%) experienced portal thrombus following graft failure. However, none of the patients were treated with PTPoA because of their severe general condition. Only one patient in the hepatic PoH group was treated via PTHA because of a suspicious anastomotic stricture of the HV.

### IVR for anastomotic vascular stricture

Pre-hepatic PoH was caused by PV complications, including PV stricture (PVS) with/without PVT in 13 patients and PVT alone in 3. PTPoA was performed in 62.5% (10/16) of patients with pre-hepatic PoH. Patencies were observed in all 10 patients after the first intervention. The procedures were repeated two to nine times in half of the cases, one of which required the insertion of a metallic stent. Patency of the PV was maintained for a mean duration of 11.5 ± 7.8 years in all patients (Table 3).

**Table 3:** Cases of IVR for PoH

Case No.	Sex	Age at 1 st IVR, years	Indication for LT	Type of graft	Years between LT and 1 st IVR	Indication for IVR	IVR procedure	Frequency of IVR	Additional procedure	Complications related to IVR	Final statement	Follow-up duration, years	Patient outcome
1	M	1.3	FHF	Lateral	0.2	HVS	PTHA	1			Patent HV	0.4	Graft loss
2	F	50.4	FAP	Left	0.3	HVS	PTHA	1			Patent HV	15.0	Alive
3	M	34.4	FAP	Left	0.3	HVS	PTHA	1			Patent HV	12.0	Alive
4	M	2.5	PNC	Lateral	2.1	HVS	PTHA	2			Obstract HV	2.6	Graft loss
5	F	3.5	FHF	Lateral	4.6	HVS	PTHA	15	Stent		Obstract HV	9.9	Graft loss
6	M	1.2	BA	Lateral	11.9	HVS	PTHA	1			Patent HV	18.1	Alive
7	M	19.6	BA	Lateral	16.7	HVS	PTHA	1			Patent PV	0.1	Alive
8	F	2.1	Alagille	Lateral	1.4	HVS and PVS	PTH and PTPoA	3			Patent HV and PV	8.0	Alive
9	M	19.2	BA	Left	0.0	PVS	PTPoA	2			Patent PV	15.9	Alive
10	F	8.7	BA	Lateral	0.1	PVS	PTPoA	1			Patent PV	2.8	Alive
11	M	5.2	BA	Lateral	0.9	PVS	PTPoA	2			Patent PV	28.9	Alive
12	F	2.9	BA	Lateral	2.2	PVS	PTPoA	2			Patent PV	11.6	Alive
13	M	3.8	BA	Lateral	3.3	PVS	PTPoA	2			Patent PV	7.2	Alive
14	M	39.7	FAP	Left	3.8	PVS	PTPoA	1			Patent PV	14.1	Alive
15	F	52.9	Citrullinemia	Left	3.8	PVS	PTPoA	1		Biliary portal fistula	Patent PV	12.6	Alive
16	F	52.5	PBC	Left	4.0	PVS	PTPoA	1			Patent PV	1.6	Graft loss (HCV)
17	M	6.0	BA	Lateral	4.5	PVS	PTPoA	9	Stent		Patent PV	17.5	Alive
18	F	28.7	Citrullinemia	Left	9.9	PVS	PTPoA	1			Patent PV	15.7	Alive
19	F	19.9	Wilson	Left	7.7	Esophageal varix	PSE	1		PVT	Obstract PV	4.3	Graft loss

BA, biliary atresia; FAP, familial polyneuropathy: FHF, fulminant hepatic failure; HCV, hepatitis C virus; HVS, hepatic vein stenosis; IVR, interventional radiology; NH, neonatal hepatitis; PBC, primary biliary cholangitis; PSE, partial splenic embolization; PTHA, percutaneous transhepatic hepatic vein angioplasty; PTPoA, percutaneous transhepatic portal vein angioplasty; PV, portal vein; PVS, portal vein stricture; PVT, portal vein thrombosis; PoH, portal hypertension

All eight patients with post-hepatic PoH had HV stenosis (HVS). Among them, percutaneous transhepatic HV balloon angioplasty was successfully performed in seven patients, and percutaneous transjugular HV balloon angioplasty was successfully performed in the remaining patient. However, repeated procedures were required in 3 (37.5%) patients, including the insertion of a metallic stent in 1. Three patients lost their graft because of graft failure 0.4, 2.6, and 9.9 years after the first IVR (Table 3). The mean patency was 8.3 ± 6.8 years. One patient experienced PVS 11 months after successful IVR for HVS and underwent PTPoA for PVS (Table 3, Case No. 8).

### Treatment for hepatic PoH

Splenectomy and/or devascularization of the shunt vessels reduces splenic flow, resulting in a reduction in portal pressure. We performed these procedures in only two patients with an insufficient evaluation owing to a relatively short follow-up period. We expect the graft to be functional; however, this requires further investigation.

Large-volume paracentesis for pleural effusion and/or ascites is a conservative treatment for symptom relief in end-stage liver disease and the only viable treatment in 15.4% of patients with hepatic PoH after LT, highlighting the difficulty in treating hepatic PoH.

**Table 4:** Results of a Cox proportional hazard regression analysis of factors predictive of graft loss after living donor liver transplantation

Variable	Univariate analysis		Multivariate analysis		
	HR (CI)	*p*-value	HR (CI)	*p*-value	
Recipient age at LT	1.99 (1.68–2.43.68.43)	<0.0001	3.46 (0.76–15.82.76.82)	0.76	
MELD	1.06 (1.01–1.11.01.11)	<0.05	3.00 (0.98–1.07.98.07)	0.31	
Presence of HCC	6.33 (1.41–28.44.41.44)	<0.05	2.96 (0.98–8.96.98.96)	0.06	
Rejection episodes	5.84 (2.11–16.18.11.18)	<0.001	4.16 (1.62–10.66.62.66)	<0.005	
Post-transplant PoH	9.00 (3.04–26.70.04.70)	<0.0005	5.73 (2.43–13.55.43.55)	<0.0005	

CI, confidence interval; HCC, hepatocellular carcinoma; HR, hazard ratio; MELD, model of end-stage liver disease; LT, liver transplantation; PoH, portal hypertension.

### Treatment outcomes in the three types of PoH

**Fig. 3:**
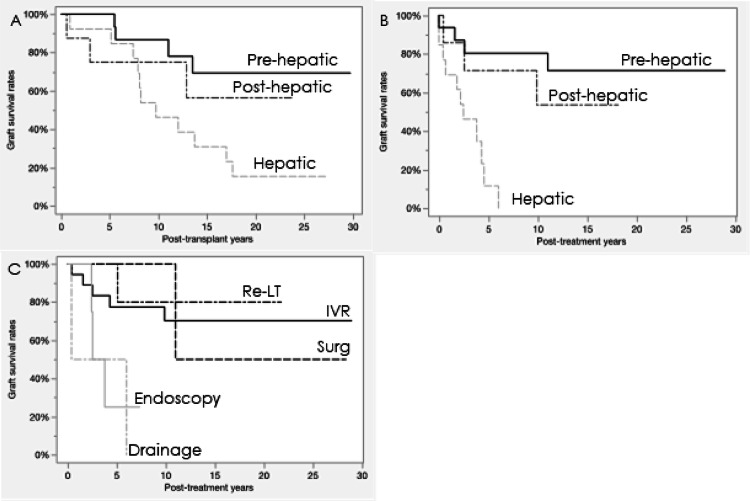
Treatment outcomes of liver transplant (LT) recipients with portal hypertension (PoH). (**a**) The graft survival rate was significantly worse in the hepatic PoH group (*n* = 13, grey solid line) than in the pre- (*n* = 16, solid black line) and post-hepatic PoH groups (*n* = 8, long dashed-dotted line). The 10-year graft survival rates were 46.2%, 86.7%, and 75.0% in the hepatic, pre-hepatic, and post-hepatic groups, respectively (*P* < 0.05). (**b**) The graft survival rates after the first treatment for post-transplant PoH were significantly better in the pre- (*n* = 16, solid black line) and post-hepatic PoH groups (*n* = 8, long dashed-dotted line) than in the hepatic PoH group (*n* = 13, grey solid line), with 10-year graft survival rates of 80.4%, 53.6%, and 0.0%, respectively (*P* < 0.0005). (**c**) Patients treated with re-transplantation (Re-LT group, *n* = 7, black long dashed-dotted line), surgical intervention (e.g. splenectomy; Surg group, *n* = 5, black dotted line), and interventional radiology (IVR group, *n* = 19, solid black line) for post-transplant PoH had significantly better graft survival rates than those treated with only drainage of pleural effusion and/or ascites (Drainage group, *n* = 4, solid grey line) and endoscopic intervention (Endoscopy group, *n* = 4, long dashed-dotted line) after the first treatment. The 10-year graft survival rates after the first treatment were 75.0%, 100.0%, 70.4%, 0.0%, and 0.0% in the Re-transplantation, Surg, IVR, Drainage, and Endoscopy groups, respectively (*P* < 0.01)

The graft survival rate was significantly worse in the hepatic PoH group than in the pre- and post-hepatic PoH groups after LT (10-year graft survival rate: 46.2% vs. 86.7% and 75.0%, respectively; *p* < 0.05) (Fig. 3a) and the first treatment for post-transplant PoH (10-year graft survival rate: 0.0% vs. 80.4% and 53.6%, respectively; *p* < 0.0005) (Fig. 3b). Re-transplantation, surgical intervention, and IVR had better graft survival rates than drainage of pleural effusion and/or ascites and endoscopic intervention after the first treatment (10-year patient survival rates: 80.0%, 100.0%, and 70.4% vs. 0.0% and 0.0%, respectively; *p* < 0.01) (Fig. 3c).

### Predictors of graft loss

A Cox regression analysis showed that recipient age at LT, MELD score, HCC presence, rejection episodes, and post-transplant PoH were associated with graft loss (Table 4). Posttransplant PoH was identified as an independent predictor of graft loss (HR: 5.73, 95% CI: 2.43–13.55, *p* < 0.0005), together with rejection episodes (Table 4).

## Discussion

In the present study, 11.4% of LT recipients experienced PoH complications that negatively affected the graft survival rate.

PoH occurs after LT under conditions of not only liver cirrhosis, but also of non-cirrhotic diseases such as metabolic liver diseases. Both pre- and post-hepatic PoH cases due to vascular complications accounted for a large proportion of PoH cases after LT. Five of six patients with metabolic liver disease developed post-transplant PoH due to vascular disease, and the remaining patient developed hepatic PoH due to liver graft cirrhosis due to *de novo* autoimmune hepatitis, suggesting that post-transplant PoH was not associated with the pre-transplant PoH condition. Pre-hepatic, hepatic, and post-hepatic PoH have unique characteristics and require different treatments. Therefore, it is necessary to determine the type of PoH after LT. The ratio of metabolic liver disease cases and, consequently, the pre-transplant MELD score was significantly different among the three groups. Post-hepatic PoH that was initially identified as ascites and/or pleural effusion in 87.5% of cases appeared as an anastomotic stricture of the HV in the early period after LT, requiring differentiation from small-for-size syndrome [7, 8]. Post-hepatic PoH was treated using IVR in all cases. A lateral segment graft was used in 75% of post-hepatic PoH cases, probably because of technical complications in hepatic vein construction in pediatric recipients. Pre-hepatic PoH that presented with various symptoms occurred as an anastomotic stricture of the PV and PVT, probably depending on the occupancy rate of the thrombus in the PV. In some cases, PVT is further complicated following hepatic PoH. Pre-hepatic PoH was mainly treated with IVR. However, in some cases, pre-hepatic PoH leads to liver graft failure, which requires re-transplantation. Hepatic PoH occurred with esophageal varices (68.8%) and portal vein thrombosis (50.0%) in the late-onset period because of graft failure caused by recurrent primary disease, rejection, and *de novo* hepatitis, suggesting slow development and existence of PoH for a long duration. Hepatic PoH is the most difficult to treat, has severe outcomes, and frequently requires re-transplantation.

Portal occlusion due to stricture and thrombosis is the most common pre-hepatic etiology of PoH after LT, with a reported incidence of 1%–6% [9–11]. Vascular complications have been reported to occur more frequently in LDLT and pediatric LT than in DDLT using full-size liver grafts, possibly because of caliber differences and torsion occurring at the vascular anastomosis caused by graft hypertrophy and recipient growth [11–13]. This study cohort included 40% pediatric patients. Therefore, the rate of pre-hepatic PoH was higher in the present study than in previous reports.

HVS occurs after LT with a reported incidence of 1%–4% [10, 14]. The incidence is higher in LT involving a living donor and other partial liver reconstructions than in whole LT, where venous reconstruction is usually performed using the piggyback technique [15]. In the present study, HVS occurred in 2.5% of cases in which a living donor partial graft was used, and all cases were successfully treated using IVR.

Regarding the treatment for post-transplant PoH, large-volume paracentesis for pleural effusion and/or ascites is a conservative treatment, regardless of disease origin. Similarly, the endoscopic variceal ligation and sclerotherapy approach is used only in cases of esophagogastric varices, which are complications associated with PoH. These treatments showed poor outcomes, indicating that PoH requires direct treatment to approach the origin. IVR, including balloon dilatation, is a promising treatment for both pre- and post-hepatic PoHs. However, most hepatic PoH cases require re-transplantation.

IVR has been widely used to treat vascular complications after LT, such as PV, HV, and IVC strictures [16]. PVS was successfully treated via PTPoA without stenting, except in one pediatric recipient who required up to nine balloon dilatations (Table 3, Case No. 17). The graft survival rate was 100%, although multiple procedures were required to maintain the graft function in half of the patients. None of the patients with PVS experienced graft failure. In contrast, HVS was initially successfully treated using IVR, but one-third of the patients ultimately experienced graft failure.

Refractory ascites is frequently found in patients with PoH after LT and is caused by various factors, such as allograft failure, rejection, recurrent liver disease, bacterial peritonitis, and vascular complications. Large-volume paracentesis palliates refractory ascites but does not treat the cause [17, 18]. Therefore, the cause of PoH should be investigated in the case of large-volume paracentesis. 15% of patients with hepatic PoH were treated using only large-volume paracentesis but could not proceed with further treatment. Pereira et al. proposed a management algorithm for refractory ascites associated with PoH after LT and recommended interventional or surgical treatment for vascular causes, such as PVS [17]. 

Treatment strategies that approach shunt vessels, such as splenectomy and/or surgical devascularization, can be used for PoH after LT. However, only a few studies have reported these treatments, and their outcomes are still unknown. Therefore, long-term investigation of our cases is required to clarify the efficacy of these procedures. Although a transjugular intrahepatic portosystemic shunt has been proposed as a bridge to LT by improving refractory ascites or variceal bleeding in patients with PoH, its efficacy in hepatic PoH after LT is unknown [19]. 

Several limitations associated with the present study warrant mention. First, this was a single-center retrospective study with a limited number of cases. Second, a definition of PoH after LT has not yet been established. Although PV pressure measurement is required to diagnose PoH, this procedure is invasive. In this study, portal pressure measurements were performed only in the cases of IVR. Therefore, PoH was diagnosed based on symptoms, such as gastrointestinal varices, hepatic hydrothorax and/or ascites, and hypersplenism. Furthermore, measurement of the hepatic venous pressure gradient via the peripheral antecubital vein might be a minimally invasive method that provides an indirect measurement of PV pressure in LT recipients [20, 21]. Since only a few reports have described PoH after LT, a multicenter study is necessary to validate these findings. Third, this study included only LDLT rather than DDLT because DDLT was performed in a limited number of cases in Japan owing to donor shortage.

In conclusion, PoH developed in 11.4% of the LT recipients and negatively affected the patient survival. IVR was able to improve the graft survival rate of post-transplant PoH in LT recipients. Pre- and post-hepatic PoH were successfully treated using IVR, but hepatic PoH was difficult to treat and frequently required re-transplantation. Establishing a diagnostic and therapeutic strategy for post-transplant PoH in LT recipients is necessary.

## Supplementary Information

Below is the link to the electronic supplementary material.

Supplementary material 1 (DOCX 21.3 kb)

Supplementary material 2 (DOCX 19.4 kb)

## Data Availability

The data supporting the findings of this study are available from the corresponding author upon reasonable request

## Abbreviations

CI: Confidence interval
DDLT: Deceased donor liver transplantation
GRWR: Graft recipient weight ratio
GV/SLV: Ratio of graft volume to standard liver volume
HCC: Hepatocellular carcinoma
HR: Hazard ratio
HV: Hepatic vein
HVS: Hepatic vein stenosis
IVC,: Inferior vena cava
IVR: Interventional radiology
LT: Liver transplantation
MELD: Model of end-stage liver disease
PoH: Portal hypertension
PTHA: Percutaneous transhepatic (or transjugular) hepatic vein angioplasty
PTPoA: Percutaneous transhepatic portal vein angioplasty
PV: Portal vein
PVT: Portal vein thrombosis
