# Supplementary material for: Characteristics and treatment outcomes of portal hypertension after living donor liver transplantation
Source: Surg Today. 2026 Jan 7;56(7):1191–200. doi: 10.1007/s00595-025-03222-8 (PMC13303323; doi:10.1007/s00595-025-03222-8)
Supplement: Supplementary file 1 — Supplementary material 1 (DOCX 21.3 kb) [file 595_2025_3222_MOESM1_ESM.docx]

Supporting Information Table 1. Characteristics of pediatric patients in the PoH and no-PoH groups

| Pediatrics, n=130 | PoH, n=21 | no-PoH, n=109 | p-value |
| --- | --- | --- | --- |
| Age at LT, years | 1.5 (0.8, 11.5) | 1.3 (0.8, 3.9) | 0.66 |
| Female, n (%) | 10 (47.6) | 71 (65.1) | 0.13 |
| Primary disease, n (%) |  |  | 0.47 |
| Biliary disease | 15 (71.4) | 92 (84.4) |  |
| Hepatocellular disease | 5 (23.8) | 14 (12.8) |  |
| Metabolic liver disease | 1(4.8) | 2 (1.8) |  |
| Others | 0 (0.0) | 1 (0.9) |  |
| HCC, n (%) | 0 (0.0) | 2 (1.8) | 0.40 |
| Child–Pugh score | 10 (8, 11.5) | 10 (8, 11) | 0.80 |
| PELD score (n=115) | 16 (11.5, 23) | 16.5 (10.3, 23) | 0.76 |
| MELD score (n=15) | 26.5 (13.8, 38.5) | 15 (13, 18) | 0.24 |
| Graft types, n (%) |  |  | 0.055 |
| Lateral segment | 14 (66.7) | 93 (85.3) |  |
| Left lobe | 7 (33.3) | 16 (14.7) |  |
| Graft volume, mL | 320.0 (249.0, 373.5) | 272.0 (240.0, 309.0) | 0.07 |
| GV/SLV, % | 81.5 (49.7, 105.7) | 82.8 (62.3, 103.0) | 0.75 |
| GRWR, % | 2.64 (1.30, 3.80) | 2.86 (1.86, 3.83) | 0.58 |
| Relationship to donor, n (%) |  |  | 0.26 |
| Parents | 20 (95.2) | 108 (99.1) |  |
| Others | 1 (4.8) | 1 (0.9) |  |
| ABO-compatibility, n (%) |  |  | 0.09 |
| Identical | 18 (85.7) | 77 (70.6) |  |
| Compatible | 1 (4.8) | 25 (22.9) |  |
| Incompatible | 2 (9.5) | 7 (6.4) |  |
| Post-splenectomy, n (%) |  |  |  |
| Operative time, min. | 759 (689, 875.5) | 713 (630, 865) | 0.26 |
| Blood loss, mL | 630 (355, 1,200) | 657 (367, 1,622) | 0.730 |
| Follow-up period, years | 13.2 (9.9, 22.8) | 23.4 (17.3, 28.2) | 0.01 |

GRWR, graft recipient weight ratio; GV/SLV, ratio of graft volume to standard liver volume; HCC, hepatocellular carcinoma; LT, liver transplantation; MELD, model of end-stage liver disease; PELD, pediatric end-stage liver disease; PoH, portal hypertension
