# Supplementary material for: Characteristics and treatment outcomes of portal hypertension after living donor liver transplantation
Source: Surg Today. 2026 Jan 7;56(7):1191–200. doi: 10.1007/s00595-025-03222-8 (PMC13303323; doi:10.1007/s00595-025-03222-8)
Supplement: Supplementary file 2 — Supplementary material 2 (DOCX 19.4 kb) [file 595_2025_3222_MOESM2_ESM.docx]

Supporting Information Table 2. Characteristics of adult patients in the PoH and no-PoH groups

| Adults, n=195 | PoH, n=16 | no-PoH, n=179 | *p*-value |
| --- | --- | --- | --- |
| Age at LT, years | 42.1 (31.4, 49.9) | 50.0 (35.5, 58.6) | 0.12 |
| Female, n (%) | 11 (68.8) | 95 (53.1) | 0.22 |
| Primary disease, n (%) |  |  | 0.76 |
| Biliary disease | 5 (31.3) | 44 (24.6) |  |
| Hepatocellular disease | 6 (37.5) | 84 (46.9) |  |
| Metabolic liver disease | 5 (31.3) | 48 (26.8) |  |
| Others | 0 (0.0) | 3 (1.7) |  |
| HCC, n (%) | 1 (6.3) | 43 (24.0) | 0.07 |
| Child–Pugh score | 9 87, 10.8) | 9 (6, 11) | 0.99 |
| MELD score | 14.5 (8.8, 19) | 14 (9, 20) | 0.65 |
| Graft types, n (%) |  |  | 0.44 |
| Left lobe | 15 (93.8) | 157 (87.7) |  |
| Right lobe | 1 (6.3) | 22 (12.3) |  |
| Graft volume, mL | 414.0 (352.5, 450.5) | 424.0 (382, 486.0) | 0.29 |
| GV/SLV, % | 38.2 (33.1, 46.0) | 38.4 (34.3, 43.9) | 0.62 |
| GRWR, % | 0.80 (0.66, 0.94) | 0.79 (0.67, 0.93) | 0.93 |
| Relationship to donor, n (%) |  |  | 0.10 |
| Parents | 3 (18.8) | 32 (18.3) |  |
| Children | 3 (18.8) | 75 (42.9) |  |
| Non-kinship | 3 (18.8) | 38 (21.7) |  |
| Others | 7 (43.8) | 32 (18.3) |  |
| ABO-compatibility, n (%) |  |  | 0.86 |
| Identical | 12 (75.0) | 131 (73.2) |  |
| Compatible | 3 (18.8) | 41 (22.9) |  |
| incompatible | 1 (6.3) | 7 (3.9) |  |
| Post-splenectomy, n (%) | 1 (6.3) | 37 (20.7) | 0.12 |
| Operative time, min. | 941 (901, 1,059) | 947 (822.5, 1,063) | 0.76 |
| Blood loss, mL | 1,055 (462.5, 4,800) | 2,275 (985, 5,200) | 0.13 |
| Follow-up period, years | 15.6 (8.7, 18.9) | 14.4 (3.6, 21.1) | 0.82 |

GRWR, graft recipient weight ratio; GV/SLV, ratio of graft volume to standard liver volume; HCC, hepatocellular carcinoma; LT, liver transplantation; MELD, model of end-stage liver disease; PoH, portal hypertension
